# A Mother's Cry: A Race to Eliminate the Influence of Racial Disparities on Maternal Morbidity and Mortality Rates Among Black Women in America

**DOI:** 10.7759/cureus.9207

**Published:** 2020-07-15

**Authors:** Geraldine I Oribhabor, Maxine L Nelson, Keri-Ann R Buchanan-Peart, Ivan Cancarevic

**Affiliations:** 1 Obstetrics and Gynecology, California Institute of Behavioral Neurosciences & Psychology, Fairfield, USA; 2 Internal Medicine, California Institute of Behavioral Neurosciences & Psychology, Fairfield, USA

**Keywords:** maternal, mortality, morbidity, minority, black, racial, disparities, pregnancy, ethnic, women

## Abstract

Racial/ethnic disparities in maternal care exist, even as medicine continues to progress on several aspects, medical care continues to fail countless women each year, particularly minority women and women of color. Black and American Indian/Alaska Native women experienced exponentially more pregnancy-related deaths. Recognizing factors that underlie disparities in pregnancy-related deaths and implementing preventive approaches to resolve them may mitigate racial/ethnic disparities in pregnancy-related mortality. Future research on these disparities should focus on strategies for reducing racial/ethnic inequalities in pregnancy-related deaths, including improving access to high-quality preconception, maternity, and postpartum care for minority women, multi-ethnic education for physicians and healthcare providers in a bid to eliminate implicit biases, adequate funding, and improvement of healthcare facilities in minority areas, education of healthcare providers on variation in the incidence of some certain conditions in different ethnic groups so that care is patient-centered and culturally appropriate. All of these can be enforced through the community, healthcare facility, patient, family, physician, and system-level collaboration.

## Introduction and background

Maternal death, described as the death of a woman during pregnancy or within 42 days of the end of a pregnancy - regardless of the result, period or place of pregnancy - from any cause linked to or caused by the pregnancy or its management, but not from unintended or incidental causes [1]. As reported by the Centers for Disease Control and Prevention (CDC), the United States maternal mortality rate (MMR) has more than doubled, from 7.2 deaths per 100,000 live births in 1897 to 16.7 deaths per 100,000 live births in 2016, while the number of reported pregnancy-related deaths has seen a steady decline in most parts of the world [2].

The pregnancy-related mortality ratio is the approximate of pregnancy-related deaths per 100,000 live births. It varies among different ethnicities. From 11.3 deaths per 100,000 live births for Hispanic women to 13 deaths per 100,000 live births for white non-Hispanic women and a staggering 42.4 deaths per 100,000 live births for black non-Hispanic women between years 2011 and 2016 [3]. With black non-Hispanic women 3.3 times more likely to die from pregnancy-related causes, it is safe to say that the sizeable racial/ethnic disparities in pregnancy-related mortality are hard to overlook.

Considering these disturbing patterns, growing emphasis is being centered on minimizing racial and ethnic contrariety in maternal morbidity and mortality in the United States. The fact that the incidence of pregnancy-related mortality of black women in some parts of the United States is close to that of women in some developing countries has concerned health practitioners, doctors, and policymakers. Most studies have shown to date that about half of significant maternal morbidity cases and maternal fatalities are preventable, rendering healthcare efficiency a crucial tool to tackle racial and ethnic inequalities in their incidence [4].

This article is aimed at identifying how the quality of medical care, especially delivery and clinical care, leads to racial inequalities in extreme maternal morbidity and mortality, the underlying causes of these inequalities, and the prospective levers to mitigate these inequalities throughout the spectrum of maternity care.

## Review

Identifying the causes of healthcare disparities

Socioeconomic Factors

Health inequality stems from social, economic, environmental, and structural disparities that contribute to differences in health outcomes among and within communities. One of the root causes of health inequity is the unequal distribution of resources and power, including commodities, facilities and social attention across different dimensions of identity, giving rise to unequal social, economic, and environmental circumstances, also called social determinants of health [5]. African Americans are at a poverty rate of 20.8%, more than doubling Non-Hispanic whites at 8.1%, combining this with pre-existing racial inequalities in the rates of health insurance coverage, differences in access to healthcare, the inclination to work low-income paying jobs with no health benefits, the limitations causing severe constraints and hardship that lead to unwanted pregnancy complications, inaccessible prenatal and perinatal care persists, posing significant health risks for the black mother and child [6,7]. Still, Black mothers face significant obstacles in receiving high-quality healthcare.

Pre-Pregnancy Care

It is well acknowledged that optimizing preconception care improves pregnancy outcomes by improving women's overall physical health and reproductive planning [8]. The high rates of obesity, hypertension, diabetes, and chronic disease among ethnic and racial minority women and the close correlation between these comorbidities and adverse maternal outcomes emphasize the critical need to focus on preconception [9,10]. Roughly half of the pregnancies are accidental, highlighting the need for strong contraceptive emphasis [11]. Unintended pregnancies have been associated with numerous adverse perinatal outcomes, and there are substantial racial/ethnic differences in unintended rates of pregnancy, with black and Hispanic women having higher rates of unintended pregnancy than white women [12].

Although there is less study of the association between unintended pregnancies with severe maternal morbidity and mortality, unintended pregnancies are associated with adverse pregnancy outcome including maternal depression, should be regarded in interventions delineated to improve maternal and child health [13].

Prenatal Factors

Early and adequate prenatal care is thought to promote healthy pregnancies by screening and managing the risk factors and conditions of a woman's health and encouraging healthy behaviors during pregnancy. Several studies have shown a link between fewer prenatal visits and poorer outcomes of pregnancy, such as low birth weight, premature birth, and infant mortality [14]. There is a little known correlation between antenatal treatment and maternal outcomes. However, data show that no or few prenatal visits are associated with maternal mortality and severe maternal morbidity [15]. The timing and receiving of prenatal care vary significantly according to race and ethnicity. In 2012, initiation of prenatal care in the first trimester was highest for white and Asian women (79% and 78% respectively), followed by mixed-race and Hispanic women (71% and 69%, respectively), and lowest for black, Native Hawaiian/other Pacific Island and American Indian/Alaska Native women (64%, 55%, and 59%, respectively) [16].

A multitude of factors plausibly play a role in the receipt and timing of adequate prenatal care, including but not limited to the high cost of care, insurance availability, commuting challenges, and lack of culturally competent care. According to a retrospective/prospective cohort study, 40% of participating subjects reported communication difficulties while one quarter reported bias during intrapartum hospital stay [17]. Nonwhite race/ethnic background was connected to almost three times greater likelihood of prejudice owing to color, dialect, or culture [17].

Quality of Hospitals

Recent evidence indicated that a significant proportion of racial and ethnic disparities in severe maternal mortality and morbidity could be explained by differences in hospital quality, comparable to a series of studies in other fields of medicine that have shown that minorities receive care in hospitals of dissimilar and lower quality than whites [18-20]. Some recent studies have shown that women of racial and ethnic minorities deliver in hospitals of different and more inferior quality than whites [21,22].

In a comparative study done to assess the effect of delivery site on disparities, it was estimated that if black women gave birth in the same hospitals as white women, nearly 1000 black women would be able to prevent severe and detrimental incidents during labor hospitalizations, decreasing the frequency of black maternal morbidity from 4.2% to 2.9% [22].

These studies indicate that location matters, and that focus is paid to improving the quality of treatment during delivery hospitalization to minimize inequalities in maternal mortality and morbidity. It is also vital to know that ethnic and racial inequalities among hospitals and in hospitals have been reported, and advances in the standard of care can tackle both drivers of inequalities.

Strategies for Disparity Reduction

As black and other ethnic and racial minority women have far less access to preconception and maternity care as well as deliver in hospitals of poorer quality, better resources are likely to generate health advantages for these groups. We also need to implement interventions tailored specifically towards racial/ethnic disparities not only to maternal health but healthcare services as well. The "Reduction of Peripartum Racial/Ethnic Inequalities Patient Safety Kit" has recently been released by the Council on Patient Safety in Women's Health Care and Alliance for innovation in maternal health (AIM Program), that includes action measures that hospitals and clinicians could adopt to reduce maternal morbidity and mortality inequalities [23]. As suggested by both the AIM kit and others, teaching clinicians and staff about racial and ethnic differences in maternal outcomes, the significance of mutual decision-making, cultural competence, and unconscious bias are essential measures in mitigating healthcare inequalities [23].

Another move towards improving the ethnic and racial minority women's experience is workers training on appropriate communication skills. AIM's cited tool kits answer these core issues in clinical treatment [23]. Implementing a disparity database that stratifies quality indicators by ethnicity and race is a valuable tool that enables healthcare facilities and organizations become aware of inequalities inside their hospitals and track their performance on quality measures for groups with a higher risk for negative outcomes [24]. There has been increasing evidence that programs that work with communities may have a beneficial effect on quality improvement and inequality reduction [25].

Contraception is an effective strategy, particularly for women with certain health problems, to reduce maternal mortality and morbidity. For instance, pregnancy with certain high-risk cardiovascular complications that confer increased risk of maternal mortality is not recommended [26]. Considering the increasing prevalence of obesity, hypertension, diabetes and chronic condition in women of racial/ethnic minorities as well as the significant association between such chronic health conditions and poor maternal outcomes, Preconception counseling is sacrosanct and critical in evaluating maternal risk factors such as obesity, hypertension, and tobacco use, for improving clinical health before pregnancy, and for monitoring the use of drugs in an attempt to ultimately prevent teratogenic exposure throughout conception and pregnancy [27-29]. Preconception care is a significant avenue for addressing inequalities in maternal morbidity and mortality.

## Conclusions

There are significant racial and ethnic disparities in maternal outcomes in the United States, and there is an immediate need to reduce inequalities. A growing body of evidence outlines the significant role systematic inequality plays in the formation of such disparities. The compounded nature of this issue and its adverse effects on maternal morbidity and mortality rates in racial/ethnic minority women, demands an indispensable multifaceted approach to decrease its overall incidence. Future expansion in eliminating disparities will necessitate a nationwide obligation to ensure health care equity via enhanced health insurance coverage, resource investment, public accountability centered on time-limited aims, and adequately provisioned quality improvement tactics that engage patients, communities, physicians, and healthcare institutions.
